# The Operative Treatment of Diphtheritic Laryngitis

**Published:** 1903-06-27

**Authors:** 


### THE OPERATIVE TREATMENT OF DIPHTHERITIC LARYNGITIS.

General practitioners as a rule can pass on operative surgery to those who are fond of it, but there are certain surgical emergencies in which the practitioner, if in charge of an outlying practice, must be prepared to act for himself. One of these is when death is impending over a child from closure of the larynx in the course of diphtheria. Formerly tracheotomy was the only recourse open, but O'Dwyer's introduction and perfection of intubation supplied an alternative. The claims of the latter to consideration have been increased by the use of anti-toxine, but the number of statistical reports of the result of its adoption in association with serum have been singularly few. The most noticeable are those of Kellock[1] and Conrad Basan,[2] published in 1896 and 1901 respectively, and to these H. A. T. Fairbank[3] has recently added a number of cases derived from his experience as resident superintendent at the Great Ormond Street Hospital for Children. Taken together the aggregate recovery percentage of these three observers is 76·9, which may be compared with a similar aggregate derived from cases published by W. P. Herringham,[4] G. Thornton,[5] and Roux.[6] In these anti-toxine was associated with tracheotomy and the percentage of recovery was 70·2. At Great Ormond Street intubation is the rule and tracheotomy the exception, the former being performed in all cases of laryngeal diphtheria which seem to call for immediate operative relief. The method adopted differs in some respects from that of O'Dwyer and the text-books. The operation is performed with the child lying down, instead of sitting on a nurse's lap with its head resting against her shoulder, having its head slightly extended over a small pillow, and arms and body being wrapped in a blanket. As a preliminary, moreover, each arm is cased in a roll of light cardboard from axilla to wrist, so that when all is over, the child while otherwise able to use its hands, cannot bend its elbow and so interfere with the tube. The assistant stands on the left side of the bed steadying the gag with his left hand and the forehead with his right. The operator stands opposite him and except for differences due to the prone position of the patient follows text-book methods. Between attempts to introduce the tube, the assistant is directed to close or remove the gag, in order that the child may more readily recover breath. In severe diphtheria the heart's action may be very weak, and fatal syncope be caused by the momentary suffocation induced by the operative procedure added to the pre-existing cyanosis. Fairbank had one such case, and Ball[7] and Turner[8] have published others. The chief impediments to successful intubation are great œdema, very thick membrane, and spasm of the glottis. The latter gives way on inspiration if the tube is kept applied to the glottis. In great œdema some little elastic pressure is necessary to squeeze the tube in, and to ensure that it is applied in the right direction while the left forefinger is slipped behind the arytenoids, the point of the tube should be held against the posterior surface of the epiglottis. The tubular character of the violent inspiration following successful intubation is unmistakable. In its absence the tube is either in the œsophagus or blocked, and should be at once removed. The size of tube selected is of great importance in ordinary cases, as well as those with very thick membrane. The mobility of too small a tube leads to its ejectment by coughing, and increases the probability of ulceration, while one too large causes persistent cough. At Great Ormond Street the size of the child rather than its age was taken as a guide, and the tubes commonly selected were, according to O'Dwyer's gauge, suitable for children from six months to two years older than the patients under operation. The relatively larger size of the male larynx may also be borne in mind. The plaited silk string was never removed on completion of intubation, but brought out of the mouth and fastened to the cheek with sticking-plaster. Its retention makes it easy for a nurse or anyone else to instantly remove the tube if need be. When the tube has been pushed well home by the left forefinger, the obturator removed, and the epiglottis is felt partly flapping over the top, the child should be laid on its side to facilitate the escape of mucus, etc., and the violent coughing soon subsides. Various authorities have prescribed nasal, rectal, and "uphill" feeding after intubation, but in most of the Ormond Street cases no difficulty was found in mouth feeding with arrowroot thickened milk. If, however, in spite of the thickening of the milk, feeding still causes coughing, nasal, or rectal feeding must be substituted. Slight rise of temperature after intubation usually occurs irrespective of the ease or difficulty attending the operation. As for the time at which the tube should be abandoned, everything depends on the condition of the child. It is generally useless to attempt to give it up in less than 36 hours. In average cases the routine should be to remove, cleanse, and replace the tube at the end of the first 24 hours, and after another 12 hours to remove it and await results. If dyspnœa recurs it should be replaced and left for 24 hours again. If after this dyspnœa continues to recur on removal the time it is left in place can be lengthened to 48 hours. As a rule the older the child the earlier can the tube be abandoned, according to Northrup,[9] and in Fairbank's cases the average time for which the tube was used was six days nine hours in children under three, and four days five hours in those over this age. In some rarer cases use of the tube has to be persisted in long after diphtheritic conditions have passed away. Brothers has related a case in which it was used for 58 days, and Fairbank had one of 35. In some cases this is due to pure nervousness on the part of the patient giving rise to glottis-spasm when the tube is left out, and in others to ulceration of the larynx having occurred. For the former soothing words are more useful than sedatives, though the latter should be tried as well as the local application of cocaine, supra-renal extract or a mixture of the two. In spasm due to ulceration tracheotomy may have to be eventually resorted to, if the child's general condition seriously deteriorates, but usually the ulceration heals while use of the tube is continued. Recurrent œdema after the tube is removed is a certain sign of ulceration, and others are tenderness over the thyroid cartilage and staining of the tube with blood. Removal of the tube by extractor forceps is always difficult, and Fairbank considers it should never be attempted. If the string gets bitten through, the tube can be expressed by pressure upwards and backwards on the trachea below the point of the tube with the child lying on its side on the edge of the bed. If sufficiently large, the tube is rarely expelled by coughing. Blocking may occur from membrane, and the nurse should be instructed to watch for the consequent dyspnœa, and withdraw the tube if it occurs. The membrane is likely to follow. It may be expected any time during the first three or four days, and especially after reintubation if due to loose membrane. Sometimes the latter flaps over the end of the tube, and this is probably the case if there is a croupy cough in spite of the tube being in place, and recurrent attacks of short acute dyspnœa. Thick tenacious mucus also leads to blocking. Such things should be watched for and met by withdrawal. The thin profuse secretion of mucus which sometimes causes great restlessness must be met by constant swabbing and raising the foot of the bed, with the head turned to one side. Fairbank's method, it will be noted, differs from the classical procedure chiefly in the use of the prone position during operation, and the after retention of the string. To this he attaches prime importance, and does not believe that it commonly causes ulceration in cases otherwise free from it. In his four fatal cases in which a post-mortem was obtained, no trace of such so-called "string ulceration" was found. Children rapidly get used to its presence in the mouth, and it is always ready for use even by an unskilled person. It is, perhaps, the fact that extractor forceps were considered necessary by O'Dwyer and are always supplied in "Intubation cases," but are very difficult to use, which has done more than anything else to prevent the operation becoming popular. To the general practitioner it is an operation which has considerable advantages. It is true that it is not always easy, but it is certainly never more difficult, after a little practice, than is tracheotomy in a small fat child. A further advantage is that anæsthetics are unnecessary, and that as it is a bloodless operation and no cutting required, the consent of parents is more easily obtained for its performance. The after-treatment called for is also of a less skilled character, all the paraphernalia of steam tents required after tracheotomy being unnecessary. It has obvious advantages over tracheotomy in cases of laryngeal obstruction complicated by pneumonia in which it may be difficult to assess the amount of dyspnœa caused by the two factors respectively. It may be noted too that Stern, quoted by Lennox-Brown with approval, showed that in children under $3\frac{1}{2}$ years intubation gave better results than tracheotomy. This was in pre-antitoxine days, and the difference now should be still more marked.

[1] Lancet, Oct. 3, 1896. [2] Idem, July 13, 1901. [3] Idem, June 20, 1903. [4] Allbutt's "System," vol. I., p. 755. [5] Lancet, July 8, 1899. [6] Idem, Sept. 22, 1894. [7] Idem, Nov. 26, 1892. [8] St. Thomas's Hospital Reps. 1889. [9] Brit. Med. Journ., vol. II., 1894.

